# The Mechanism behind Influenza Virus Cytokine Storm

**DOI:** 10.3390/v13071362

**Published:** 2021-07-14

**Authors:** Yinuo Gu, Xu Zuo, Siyu Zhang, Zhuoer Ouyang, Shengyu Jiang, Fang Wang, Guoqiang Wang

**Affiliations:** Department of Pathogeny Biology, College of Basic Medical Sciences, Jilin University, Changchun 130021, China; guyn20@mails.jlu.edu.cn (Y.G.); zuoxu18@mails.jlu.edu.cn (X.Z.); zsy20@mails.jlu.edu.cn (S.Z.); oyze@mails.jlu.edu.cn (Z.O.); jsy20@mails.jlu.edu.cn (S.J.)

**Keywords:** influenza virus, cytokine storm, age, sex, obesity

## Abstract

Influenza viruses are still a serious threat to human health. Cytokines are essential for cell-to-cell communication and viral clearance in the immune system, but excessive cytokines can cause serious immune pathology. Deaths caused by severe influenza are usually related to cytokine storms. The recent literature has described the mechanism behind the cytokine–storm network and how it can exacerbate host pathological damage. Biological factors such as sex, age, and obesity may cause biological differences between different individuals, which affects cytokine storms induced by the influenza virus. In this review, we summarize the mechanism behind influenza virus cytokine storms and the differences in cytokine storms of different ages and sexes, and in obesity.

## 1. Introduction

The COVID-19 pandemic showed the catastrophic impact of a new type of virus on human health. Since the H1N1 Spanish influenza outbreak in 1918, there have been many influenza pandemics, such as the H2N2 Asian influenza in 1957, the H3N2 Hong Kong influenza in 1968, and the H1N1 influenza pandemic in 2009 [1]. Between 1997 and 2014, the cross-species spread of avian influenza viruses (e.g., H5N1, H7N9, and H10N8) broke out [2,3]. Influenza-virus infection is the most common cause of lung-injury-related deaths, and during a pandemic, influenza viruses cause higher mortality [4,5].

A mature influenza virus is wrapped by a protein membrane that contains two antigenic determinants: Hemagglutinin and neuraminidase (HA and NA) [6,7,8,9,10]. Mutations in HA and NA are called antigenic drift, resulting in new influenza-virus strains. When an influenza pandemic occurs, interactions between humans and animals infected with different strains are common, mainly poultry and pigs. When a cell is infected with two or more different viral strains, the gene fragments are easily interchanged, which is called an antigenic shift [11,12,13,14,15]. The HA protein binds to sialic acid residues expressed in the airway or alveolar epithelium, triggering the endocytosis of viral particles [16]. The virus completes the assembly, budding, and scission on the cell membrane [17]. NA cuts the connection between the HA of newly formed viral particles and sialic acid receptors on the cell surface, releasing progeny viruses, which then infect neighboring cells or leave the individual through respiratory droplets [17].

Although it is easy to associate the severe consequences of influenza-virus infection with viral load, the host’s inflammatory response to the influenza virus is more related to the lung injury induced by the influenza virus [18,19,20,21]. The influenza virus first infects the upper respiratory tract, enters epithelial cells through endocytosis, and infects the lower respiratory tract as the disease worsens [22,23]. The influenza virus infects epithelial cells, endothelial cells, and alveolar macrophages to produce the first wave of cytokines; then, adaptive immune cells are activated and regulated to secrete the second wave of cytokines that promote viral clearance [23]. If excessive production of proinflammatory cytokines leads to aggressive proinflammatory responses and the insufficient control of anti-inflammatory responses, this series of events is called a cytokine storm, which is one of the reasons for the increased mortality during influenza-virus infection [24,25,26,27]. The cytokine storm induced by the influenza virus can lead to major immunopathology and serious disease consequences [24,28,29,30].

A severe cytokine storm can cause acute respiratory distress syndrome (ARDS) [31]. Clinically, the characteristic alveolar changes of influenza-virus pneumonia caused by cytokine storms include capillary thrombosis, focal necrosis and congestion of the alveolar wall, inflammatory infiltration, hyaline membrane formation, and pulmonary edema [32]. In severe influenza pneumonia, small-blood-vessel thrombosis, bleeding, and diffuse alveolar damage can be observed, indicating coagulation disorders [32,33,34,35,36]. The aggressive immune response of patients with a cytokine storm is enhanced by coagulation dysfunction, which is manifested by the activation of pulmonary endothelial cells, vascular leakage, diffuse intravascular coagulation, and pulmonary microembolisms [34,37,38]. Severe cytokine storms also lead to multiple organ dysfunction syndromes, systemic inflammation, and even death [31,39,40].

Although the influenza B virus (IBV) is similar to the influenza A (IAV) virus in morphology, the influenza A virus has a variety of hosts, prone to antigenic drift and antigenic shift, and a variety of epidemic strains. The influenza B virus, on the other hand, has only two hosts, humans and seals, and there are only two epidemic strains, Victoria and Yamagata. The influenza B virus is more likely to infect children [41,42]. The cytokine storm and clinical manifestations caused by the influenza B virus in children are similar to those caused by the influenza A virus. However, due to the insufficient sample size, it is not enough to rule out important differences. For the rest of this review, unless otherwise specified, we only discuss the influenza A virus. In this review, we mainly focus on the mechanism behind the dysregulation of the cytokine storm in the influenza virus.

## 2. Cytokines

**Figure 1:**
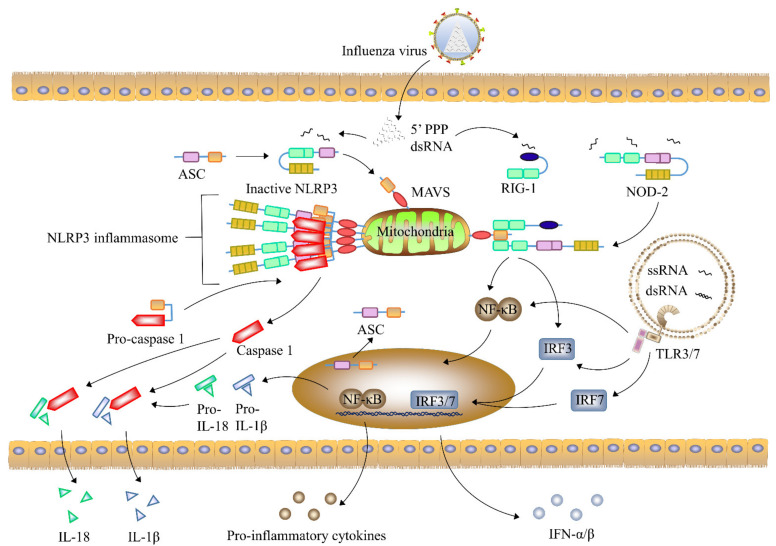
Immune pathways triggered by influenza-virus infection. Influenza viruses first infect alveolar or airway epithelial cells. Viral RNA in the cytoplasm is recognized by RIG-I, TLR3, TLR7, and NLR. TLR pathway activates downstream IRF3 and IRF7 to regulate the production of IFNs. Binding viral RNA to the RIG-I receptor can trigger its interaction with MAVS to activate NF-κB. MAVS and NLR activate inflammasomes under the action of viral RNA to release IL-1β and IL-18, thereby forming a cytokine storm.

The center of the cytokine storm is the cytokine. Cytokines are small proteins secreted by cells that are essential for the communication between cells in the immune system and the resolution of infectious diseases. Since the influenza virus is a single-stranded negative-sense RNA virus, viral replication requires viral RNA polymerase to produce sense messenger RNA from the viral genome [17]. The presence of viral RNA in the cytoplasm activates three immune pathways: Retinoic acid-induced gene-1 (RIG-I) protein, Toll-like receptors (TLRs, mainly TLR3 and TLR7), and Nod-like receptors (NLR), which initiate the innate immune response to the influenza virus [43]. The binding of viral RNA to the helicase domain on RIG-I can trigger its interaction with mitochondrial antiviral-signaling protein (MAVS). MAVS induces the production of Type I and III interferons and activates the nuclear-factor kappa-light-chain enhancer of activated B cells (NF-κB) [44]. Type I interferons produced by epithelial and endothelial cells, and alveolar macrophages upregulate the expression of many interferon-stimulating genes (ISGs) and initiate downstream antiviral responses [45]. Viral RNA activates inflammasomes through MAVS and NLR, releasing IL-1β and IL-18 [46,47]. In the adaptive immune response stage, different subgroups of T cells and Type 2 innate lymphoid cells (ILC2) are activated and regulated. These reactions all promote viral clearance. If the reaction is too intense, proinflammatory cytokines are excessively produced, and an uncontrolled cytokine storm is formed, which causes organ damage, systemic inflammation, and even death (Figure 1) [48,49,50,51].

### 2.1. Type I Interferons

There are three types of interferons (Types I–III), defined by their receptor specificity [23]. Type I interferon (IFN-α/IFN-β) was first discovered, named for its ability to interfere with viral replication [52]. During the cytokine storm induced by influenza-virus infection, Type I interferon is mainly produced by macrophages, alveolar cells, dendritic cells (DCs), and inflammatory monocytes [43,53]. Type III interferons include IFN-λ1 (IL-29), IFN-λ2 (IL-28A), IFN-λ3 (IL-28B), and IFN-λ4, which are produced by myeloid and epithelial cells, such as DCs [54]. Type III interferons perform similar functions to those of Type I interferons through the Janus kinase-signal transducer and activator of transcription (JAK-STAT) pathway [55]. IFN-γ is the only Type II interferon, mainly produced by T and natural-killer (NK) cells [56]. This binds to Type II IFNs receptors (IFNGR1 and IFNGR2) and upregulates the expression of ISGs [57].

After the influenza virus infects the lungs, Type I IFNs are rapidly expressed in various myeloid and parenchymal cells [58,59]. Although Type I IFNs play an important role in the antiviral response, if IFN-α/β is overproduced to form a cytokine storm, it mainly aggravates the disease in two ways, by hindering the control of viral immune suppression and amplifying inflammation [50,53,60]. IFNα/β can bind to the Type I IFNs heterodimeric transmembrane receptor complex, IFNα receptor (IFNAR). IFNAR is widely present in immune cells, so the IFN-α/β reaction may lead to immunopathology during the cytokine storm [61]. The tumor necrosis factor (TNF)-related apoptosis-inducing ligand (TRAIL) and its receptor, death receptor 5 (DR5), play an important role in the excessive production of Type I interferon, leading to exacerbation of ARDS [21,62,63]. Influenza-virus infection relies on protein kinase R- (PKR-) and NF-κB to induce the release of IFN-β from alveolar macrophages. IFN-β in the cytokine storm induces alveolar macrophages to express proapoptotic factor TRAIL to promote alveolar epithelial-cell apoptosis [64]. After being infected with the influenza virus, compared with wild-type (WT) mice, 129 mice lacking IFN-α/β receptors had lower morbidity and mortality. In terms of lung injury, the lungs of WT mice had a high degree of inflammatory cell infiltration, resulting in massive obstruction of the alveolar space. IFN-α/βR-/- mice had less lung infiltration, and it only existed around the bronchial tubes and blood vessels. TRAIL was less expressed on the inflammatory monocytes of IFN-α/βR-/- mice. Excessive IFN-α/β can lead to serious consequences. The reason may be that a large number of highly reactive plasmacytoid dendritic cells (pDCs) produce too much IFN-α/β, which, in turn, leads to uncontrolled inflammation and lung epithelial damage mediated by the TRAIL-DR5 interaction over time [50]. As the first line of defense against influenza-virus infection, Type I and III IFNs are a double-edged sword.

### 2.2. Type III Interferons

IFNλs or Type III IFNs share homology, expression patterns, signaling cascades, and antiviral functions with Type I IFNs [55,65]. Similar to Type I IFNs, Type III IFNs in a cytokine storm affect the proliferation of epithelial cells. The interferon receptor composed of IFNLR1 and the shared IL-10R2 chain is mainly expressed in the epithelial-cell barrier. Therefore, IFN-λ acts on lung epithelial cells, which may damage the lung barrier function, and make the host vulnerable to secondary bacterial infection and cause death [66]. Excessive IFN-λ interfered with lung repair in mice infected with the influenza virus. Compared with WT mice, Ifnlr1-/- mice have significantly reduced IFN-λ production, can resist *Staphylococcus aureus* infection, and significantly improve the proliferation of lung epithelial cells [66]. Higher levels of Type III IFNs were also detected in the bronchoalveolar lavage fluid (BALF) of patients with severe influenza. This may have been because the p53 protein induced by IFNs directly inhibited the proliferation and differentiation of epithelial cells, thereby aggravating the disease and susceptibility to bacterial superinfections [61]. In clinical practice, if IFNλs is used as a therapeutic agent for influenza-virus infection, its duration needs to be considered to avoid causing a cytokine storm.

### 2.3. Type II Interferons

NK cells produce Type II IFN, IFN-γ, in the early stage of influenza-virus infection. In the subsequent immune response, T cells become the main producer of IFN-γ [67]. T cells are activated in the local draining lymph node by migratory CD103^+^ and CD11b^+^ DCs carrying viral antigens [68]. Once activated, T cells differentiate into antigen-specific effector T cells [69]. Influenza-specific effector CD8^+^ T cells can produce cytokines, including IFN-γ and TNF-α, through a variety of antigen-dependent pathways [70]. Although CD8^+^ T cells are important for clearing the influenza virus, if the CD8^+^ T-cell response is unregulated, it can cause considerable lung disease. IFN-γ produced by CD8^+^ T cells promotes the release of lung epithelial chemokines, leading to inflammatory cell infiltration, and aggravating lung pathology and thymocyte apoptosis [71,72]. Anti-IFN-γ treatment with monoclonal antibodies against IFN-γ improved the symptoms and survival rate of mice infected with the influenza virus because the neutralization of IFN-γ can reduce pulmonary hemorrhage and inflammatory cell infiltration [73]. IFN-γ can also work with TNF-α to damage lung epithelial cells that are not infected by the influenza virus [74].

### 2.4. Tumor Necrosis Factor-α

TNF-α is almost everywhere in the body. Macrophages, monocytes, NK cells, endothelial cells, epithelial cells, and T and B lymphocytes can all express and produce TNF-α [75]. TNF-α can mediate classical proinflammatory signaling pathways NF-κB and mitogen-activated protein kinase (MAPK) through TNF receptor 1 (TNFR1) and TNF receptor 2 (TNFR2) to promote downstream inflammation cascades and leukocyte infiltration [76,77,78]. Excessive TNF-α activates the signal transducer and activator of transcription 3 (STAT3) pathway through NF-κB-mediated IL-6 [79]. Uncontrolled TNF-α production leads to the excessive activation of NF-κB, MAPK, and STAT3 signaling pathways, followed by excessive production of cytokines and chemokines, forming a cytokine storm [28]. As a typical proinflammatory factor, TNF-α is located in the center of the cytokine storm [80]. Similar to IFN, TNF-α can also damage the endothelial barrier, and cause pulmonary edema and tissue damage [81]. TNF-α inhibits the key tight-junction protein claudin-5 through NF-κB, leading to capillary leakage, a large amount of plasma protein flow, and white blood cells that infiltrate the surrounding tissue [82,83]. Compared with H1N1 and H3N2, H5N1 can induce more TNF-α [84]. Compared with WT mice, TNFR-deficient mice are more resistant to fatal H5N1, survive an average of 2 days longer, and have lower levels of cytokines in the lungs, including IFN-γ and interleukins (ILs) [85,86]. Influenza patients generally seek medical attention only after they have symptoms such as fever and cough. Therefore, while inhibiting viral replication, controlling TNF-α levels to reduce the cytokine storm is a potential strategy to reduce lung pathology.

### 2.5. Interleukin-1β and Interleukin-18

The name interleukin originally referred to the cytokine secreted by leukocytes, but many kinds of cells can produce interleukin. Inactive precursor cytokines pro-IL-1β and pro-IL-18 require nucleotide-binding domain, leucine-rich-containing family, and pyrin domain-containing-3 (NLRP3) inflammasomes to mature into biologically active IL-1β and IL-18 [87]. In the process of influenza-virus infection, the activation of NLRP3 requires two signals [88]. The first signal is to activate inflammatory transcription factor NF-κB through various pattern-recognition receptors (PRRs), thereby upregulating the synthesis of pro-IL-1β, pro-IL-18, NLRP3, and caspase 1. During the second signal, NLRP3 induces the formation of a supramolecular signaling inflammasome complex via the recruitment of an adaptor apoptosis-associated specklike protein containing a CARD domain (ASC), leading to the maturation and secretion of IL-1β and IL-18 [89]. IL-1β and IL-18 bind IL-1 receptor 1 (IL-1R1) and IL-18 receptor (IL-18R) to induce NF-κB-dependent inflammation [90]. IL-18 is a proinflammatory cytokine that mediates IFN-γ production in T and NK cells [91]. IL-1β increases the transport of neutrophils and T cells to the site of infection and induces epithelial and endothelial cells to produce a second wave of cytokines, such as TNF-α and IL-6. Excessive IL-1β can aggravate the disease and cause serious consequences when H1N1, H3N2, and H7N9 are infected. Whether in the early or late stages of H1N1 or H3N2 infection, the use of targeted anti-IL-1β antibody therapy can reduce lung inflammation and improve survival [87,92]. In the face of the H7N9 challenge, NLRP3-/- and caspase1-/- mice have a higher survival rate than that of WT mice. Inflammatory cell infiltration in the lungs of WT mice is more serious, the alveolar septum is obviously thickened, part of the alveolar structure is destroyed, and the lung septum is ruptured. NLRP3-/- and caspase1-/- mice detected lower IL-1β because the absence of caspase-1 reduced the recruitment of proinflammatory cells to the lung during H7N9 infection. ASC-/- and IL-1R1-/- mice also had less lung inflammation and a higher survival rate during H7N9 infection [93]. IL-1β and IL-18 play a complex role in the cytokine storm triggered by the influenza virus. They are not only an important part of themselves but also regulate the production of TNF-α and IL-6.

### 2.6. Interleukin-6

IL-6 as a marker of inflammation is produced by macrophages, DCs, mast cells, and other innate immune cells. Clinical studies revealed that excessive IL-6 is closely related to the poor prognosis of influenza patients [18,94,95]. There are two types of IL-6 receptors (IL-6R): Membrane-bound IL-6R (mIL-6R) and soluble IL-6R (sIL-6R) [96]. mIL-6R is mainly expressed on neutrophils, monocytes, and T cells [97]. sIL-6R is produced by the limited proteolysis of membrane-bound proteins or translation of alternatively spliced mRNA [98]. H3N2 infection induces an increase in the expression of sIL-6R, and the expression of IL-6 during influenza-virus infection depends on sIL-6R [99]. This is the first report that cytokine receptors can induce cytokine expression. The influenza virus can significantly induce the expression of the suppressor of cytokine signaling 3 (SOCS3) and IL-6, both in vivo and vitro [100]. After IL-6 binds to the receptor complex containing gp130 and IL-6R/sIL-6R, STAT3 is phosphorylated by the JAK kinases [101]. The SOCS3 protein inhibits STAT3 by competing with JAKs-phosphorylated tyrosine residues, promotes the ubiquitination and degradation of the receptor–JAK–STAT complex, and inhibits JAK activities [102,103]. Compared with WT mice, SOCS3-/- mice had significantly reduced IL-6 levels after being infected with the influenza virus, returning to normal levels, preventing the production of cytokine storms [100]. IL-6 plays an important role in the cytokine storm caused by the influenza virus and provides a new target for immunotherapy strategies.

### 2.7. Interleukin-17

The IL-17 family includes at least six cytokines: IL-17A (IL-17), IL-17B, IL-17C, IL-17D, IL-17E (IL-25), and IL-17F. IL-17A and IL-17F have the highest homology in the IL-17 family, and both can transmit signals through IL-17RA [104]. Both are produced by γδ T cells after influenza-virus infection [105]. CD4+ T helper (Th) cells characterized by the expression of IL-17 are also called TH17 cells [106,107,108,109]. In addition to Th17 cells, IL-17 can be secreted by a variety of cells, such as γδ T and natural-killer cells [110,111]. IL-17 can exacerbate the inflammatory response during viral infection [112]. Th-17 hypercytokinemia is the early host response of severely ill patients with 2009 H1N1 [113,114]. IL-17 binds to a heterodimeric IL-17RA/C receptor to activate activator protein 1 (AP-1) and NF-κB signaling pathways [115,116]. The elevated expression of IL-17 in the serum of 2009 H1N1 patients may be related to the poor prognosis of the disease. Compared with WT mice, IL-17A-deficient mice have a significantly improved survival rate and reduced lung leukocyte infiltration. The survival rate of mice receiving anti-IL-17A monoclonal antibody treatment improved, and pulmonary edema also improved [117]. Influenza-virus infection activates interferon regulatory factor 3 (IRF3), NF-κB, and STAT1 to mediate the production of IFN-λ. By combining with IL-17RA, IL-17A enhances the expression of SOCS1 and SOCS3, inhibits the JAK–STAT1 pathway, which is a downstream signal effector of IFNLR, and reduces the phosphorylation of STAT1. This eventually leads to the weakening of IFN-λ induced by the influenza virus, an insufficient antiviral response, and increased inflammation [118]. After IL-17RA knockout mice had been infected with the influenza virus, the migration of neutrophils was reduced, there was only mild inflammation, the lung parenchyma was relatively unaffected, and morbidity and mortality were lower [105]. In summary, IL-17 can mediate fibroblasts or epithelial cells to produce more proinflammatory cytokines and is closely related to the cytokine storm that the influenza virus has.

## 3. Biological Factors

Numerous biological factors lead to biological differences between different individuals, which are very important in experimental studies of immunology and virology. Biological factors such as sex, age, and obesity may affect the host’s susceptibility to the influenza virus and their autoimmune response.

### 3.1. Age

Among hospitalized patients with influenza, those younger than 5 years old and older than 65 years old are more likely to develop acute pneumonia [119,120,121,122]. Therefore, it is more important for people younger than 5 years old and older than 65 years old to be vaccinated against influenza [123]. The immune response of young individuals may be more vigorous, and it is easy to trigger a stronger cytokine storm. Contrary to the overactive immune system of adolescents, the immune system of the elderly is already aging and cannot effectively respond to an influenza-virus infection in time, leading to serious pathological consequences. The different effects of the cytokine storm on the host are the main factors of serious consequences mediated by the influenza virus. Children infected with the influenza virus have similar viral burdens as those of adults [124,125]. The difference in viral titer and airway size between children and adults has nothing to do with the serious consequences of influenza [18,126,127]. Juvenile mice infected with the influenza virus had higher mortality and lung pathology than adult mice did [128], even in adult mice that were infected with twice the dose of influenza virus than that of juvenile mice [129]. Type I IFNs of juvenile mice infected with the influenza virus continued to increase. Type I IFNs can increase the production of monocyte chemoattractant protein 1 (MCP-1), which is the main chemotactic factor responsible for the recruitment of inflammatory monocytes to the lungs during infection [130,131,132]. The monocytes recruited by MCP-1 partly regulate inflammation through NLRP3 [51,133]. So, juvenile mice have more alveolar damage and immune cell infiltration than adult mice do [129]. The serious consequences of the influenza virus infection in children are more related to the cytokine storm than the viral load. This also explains why antiviral drugs such as neuraminidase have limited effects on children [134]. Therefore, the most important goal to treat children with influenza-virus infection is to control the cytokine storm.

### 3.2. Sex

During influenza-virus infection, the individual’s gender and hormonal status can regulate the process of inflammation and immunopathology [135,136]. Although different sexes have similar viral titers, women of a childbearing age experience a more severe disease than men do after being infected with the influenza virus, with more severe lung inflammation and a higher likelihood to form cytokine storms [137,138,139,140]. Natural fluctuations during menstruation, pregnancy, and menopause, or changes in hormonal concentration caused by oral contraceptives or hormone replacement therapy, can affect the trend of lung disease [141,142]. Estrogen, mainly 17β-estradiol (E2), regulates the functions of a variety of immune cells, including macrophages, DCs, granulocytes, and lymphocytes [143]. Low-dose or periodic E2 enhances the proinflammatory cytokine response and forms a cytokine storm [144]. Among H7N9 and H5N1 infections, female mortality is higher [145]. In men, testosterone is the main sex hormone. Female mice treated with testosterone had a higher survival rate in 2009 H1N1 infection, reduced IL-1β in the lungs, suppressed cytokine storm, reduced alveolar destruction, and reduced inflammatory monocyte infiltration [146]. The different effects of sex hormones on the cytokine storm caused by influenza viruses provide ideas for the development of new therapeutic strategies.

### 3.3. Obesity

Approximately 1.9 billion people in the world are overweight, and there are more than 670 million obese adults and more than 100 million obese children [147,148]. So far, no country can stop the increase in obesity rates [149]. Obese children and adults experience higher morbidity and mortality during influenza-virus infection, and the risk of secondary bacterial infections and ARDS is also increased [150,151].

The innate and adaptive immunity of obese individuals changes [152,153]. Obesity increases the accumulation of fat in bone marrow, and the development of immune cells in bone marrow is affected [154]. Both resident and circulating immune cells are affected by obesity. The total number of leukocytes and monocytes in the blood of obese individuals is more than that of thin individuals [155]. Peripheral blood mononuclear cells (PBMCs) differentiate into macrophages remaining in the tissue. As the inflammation of adipose tissue increases, macrophages transform into the proinflammatory M1 type [156]. Adaptive immune cells are also affected by obesity [157]. An increase in Th1 and Th17, and the depletion of regulatory T cells (Tregs), lead to increased immune cell infiltration and inflammation [158]. Visceral obesity attracts more B cells to interact with T cells, which is a feature of long-term low-degree inflammation [159,160]. The systemic proinflammatory state extends to the lung microenvironment, which makes it easier to form a cytokine storm.

Obese individuals have higher long-term concentrations of leptin (a proinflammatory adipokine), while adiponectin (an anti-inflammatory adipokine) is at a lower concentration [161]. Leptin is associated with elevated free fatty acid levels in obese individuals [162]. Elevated free fatty acids activate TLRs, induce monocytes to produce inflammatory cytokines, and enhance T-cell-mediated inflammation [163,164,165,166]. Adiponectin reduces macrophage activation and proinflammatory cytokine production [167]. The higher concentrations of TNF-α and IL-6 in obese patients lead to the accumulation of macrophages. These cytokines are mainly produced by internal organs and subcutaneous adipose tissue [168,169,170]. In the early stage of influenza-virus infection, IL-6, TNF-α, and Type I IFNs in high-fat-diet-induced obese (DIO) mice were delayed and decreased compared with in low-fat-diet (LN) mice [171]. In the late stage of infection, the level of inflammation in DIO mice surpassed that of LN mice [172,173]. DIO mice had more severe lung damage and inflammation, and higher mortality [174]. In DIO mice, the expression of SOCS1 and SOCS3 mRNA in the lung is upregulated, which may lead to an increase in systemic cytokine levels after influenza-virus infection, forming a cytokine storm [175]. In the late stage of influenza-virus replication, the onset of apoptosis leads to metabolic imbalance, an increase in glycolysis rate, and a decrease in adenosine triphosphate (ATP) production [176]. This influenza-virus-mediated metabolic imbalance may cause patients to become obese and provide favorable conditions for the formation of cytokine storms. The mitochondrial pyruvate dehydrogenase complex (PDC) is an important regulator of energy and metabolic homeostasis. The state of insulin resistance, including obesity, is related to the phosphorylation and inactivation of skeletal-muscle pyruvate dehydrogenase kinase (PDK). PDC links glycolysis with the tricarboxylic acid cycle (TCA) and fatty acid synthesis by catalyzing the oxidative decarboxylation of pyruvate. In mice infected with the H1N1 virus, PDC activity and ATP levels were significantly downregulated, and PDK4 expression in the skeletal muscle, heart, liver, and lungs was selectively upregulated [177]. The oral administration of dichloroacetate (DADA, a PDK4 inhibitor) to mice could restore PDC activity and ATP levels, improve metabolic disorders, and inhibit the cytokine storm [178]. The maturation, differentiation, and function of immune cells are all affected by obesity. Systemic microinflammation caused by obesity makes it easier to form a cytokine storm during influenza-virus infection. Because the immune system of obese hosts is more fragile, the damage caused by cytokine storms is often more serious.

## 4. Conclusions

The influenza pandemic poses a huge threat to human health. Many severe influenza patients die from serious complications caused by cytokine storms, such as ARDS. Endothelial dysfunction can also cause inflammation, which potentially impacts the cytokine storm caused by influenza viruses [179]. In this review, we summarized the immune mechanisms and pathways behind cytokines in the cytokine storm induced by the influenza virus. We also examined the differences of cytokine storms in different ages, sexes, and obese influenza patients. The arduous task in the future is to develop anti-inflammatory drugs targeting different biological factors from these preclinical and clinical findings. This is used to suppress the severe reaction of influenza-virus patients, control it to a normal level, and avoid the formation of a cytokine storm. Unlike antiviral drugs that target viral replication, anti-inflammatory drugs do not develop resistance, providing a new target for the treatment of influenza patients. Therefore, it is necessary to consider the combined use of anti-inflammatory drugs and antiviral drugs for the treatment of influenza patients.

## Data Availability

Not Applicable.
